# Chronic granulomatous disease: lessons in cell biology from monogenic immunodeficiency

**DOI:** 10.1093/cei/uxaf031

**Published:** 2025-05-15

**Authors:** Paige M Mortimer, Shuli Svetitsky, David C Thomas

**Affiliations:** Centre for Inflammatory Disease, Department of Immunology & Inflammation, Imperial College, London, UK; The Francis Crick Institute, London, UK; Cambridge Institute for Therapeutic Immunology and Infectious Disease University of Cambridge, UK; Department of Medicine, University of Cambridge, UK

**Keywords:** reactive oxygen species, host-pathogen interactions, immunodeficiency diseases, neutrophils, macrophage

## Abstract

Reactive oxygen species (ROS) are produced in immune cells by the phagocyte NADPH oxidase (NOX2) system that carries out coordinated transfer of electrons to molecular oxygen. The importance of the system in host defence and immunoregulation is underlined by chronic granulomatous disease (CGD), a severe monogenic immunodeficiency caused by mutations in genes encoding individual components of NOX2. CGD also leads to inflammatory manifestations due to the regulatory role of ROS in multiple signalling processes. We describe the system in detail, from its discovery to our current understanding of the oxidase. We also describe CGD and illustrate how recent insights into this disease shed light on physiology.

## Introduction

Reactive oxygen species (ROS) are vital to normal immune function. ROS such as hydrogen peroxide (H_2_O_2_) can be produced as a by-product during metabolic reactions within many cellular compartments, including mitochondria, peroxisomes and the endoplasmic reticulum. However, several homologous enzymes exist to produce ROS through the co-ordinated transfer of electrons to molecular oxygen, generating superoxide. Superoxide is then dismutated to H_2_O_2_ and other ROS such as hyprochlorous acid. This can take place at the extracellular membrane or in intracellular compartments.

ROS facilitate killing of microbes in immune cells, in concert with other systems such as neutrophil granule proteases and myeloperoxidase. ROS have an additional very important physiological function: the modulation of cell signalling. This latter function occurs when the hydrogen peroxide (H_2_O_2_) that is generated drives reversible oxidation of cysteine and methionine residues. This process modulates the activity of numerous pathways involved in immunity, including type 1 interferon, NF-κB signalling, antigen presentation, and the inflammasome. However, while the post-translational modification of amino acids such as cysteine can be utilised as a “molecular switch”, prolonged exposure of proteins and lipids to reactive oxygen species can cause irreversible damaging changes and drive pathology [1–3]. The cell’s decision to make ROS must take into account the energy cost and potential harm, and, as such, this is a tightly regulated process.

**Figure 1:**
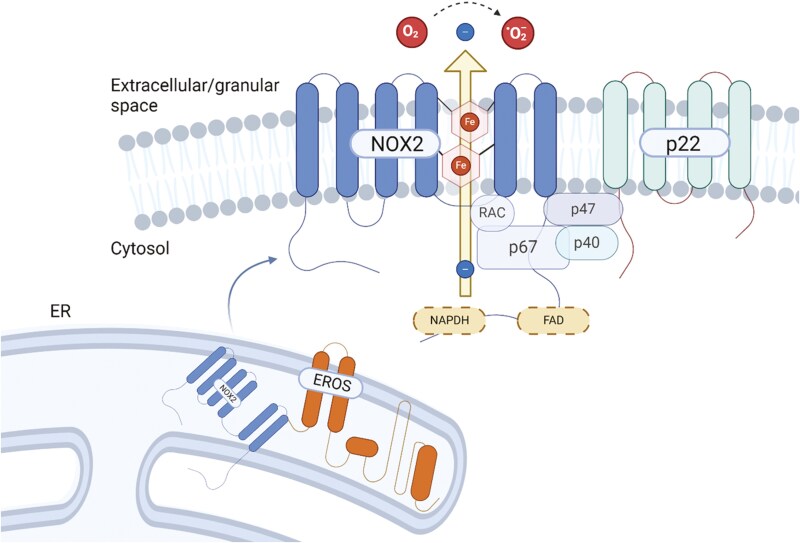
the phagocytic NADPH oxidase. The membrane-bound heterodimer consisting of NOX2 (gp91*phox*) and p22*phox*, known as cytochrome b588, utilizes a trans-membrane electron transfer system to produce ROS. The electrons are transferred from NADPH, to FAD, then are bound to heme groups and transferred to molecular oxygen. Prior to exiting the ER, NOX2 is bound to the chaperone protein EROS. Upon activation, the cytosolic components p67*phox*, p47*phox*, p40*phox* and either Rac1 (macrophages) or Rac2 (neutrophils) bind to the membrane and to the membrane-bound components, and are necessary for oxidative activity. Created in BioRender. Svetitsky, S. (2025) https://BioRender.com/u85u948

ROS are generated in immune cells by the multi-subunit phagocyte NADPH oxidase (NOX2) (Figure 1).

In this review, we set out some of the key milestones in the discovery of the oxidase and how it functions. We detail some key features of the individual subunits before describing the process of activation of the system and how this can be modulated by events. We then describe recent advances in understanding the NADPH oxidase system, including the discovery of a new essential protein—EROS. We conclude with a brief precis of chronic granulomatous disease.

## Discovery of the phagocyte NADPH oxidase and chronic granulomatous disease

In 1932, Baldridge and Gerard, studying canine neutrophils, noted an interesting phenomenon, namely that phagocytosis of bacteria was accompanied by marked uptake of oxygen [4]. An intriguing aspect of this process was that the oxygen was clearly not being used for oxidative phosphorylation because inhibitors of mitochondrial respiration had no effect on the process. However, inhibitors of glycolysis did impair it. Sbarra, Karnovsky, and colleagues extended these findings to show that “this burst of extra respiration” was accompanied by glucose consumption via the hexose monophosphate shunt and lactate production [5]. A further major advance was the observation that the uptake of oxygen could be used to make hydrogen peroxide (H_2_O_2_), as evidenced by the fact that formate is effectively converted to CO_2_ during the respiratory burst [6]. This catalytic oxidation of formate must be due to the production of substantial amounts of H_2_O_2_. This was formally validated in subsequent work through direct measurement of H_2_0_2_ production [7, 8].

The finding that neutrophils made H_2_O_2_ for host defence was elaborated by Klebanoff and colleagues, who demonstrated that the H_2_O_2_ could react with halide ions such as chloride to produce the anti-bacterial compound hypochlorite (HOCL). Further, this was catalysed by myeloperoxidase (MPO), which is abundant in neutrophil granules. Thus, phagocytosis drives both MPO release and the production of H_2_O_2_ and, consequently, the generation of hypochlorite [9].

The work described above built a narrative that neutrophils transferred electrons to oxygen for anti-microbial defence, prompting intense interest in the nature of the system that drove this process. Bernard Babior’s laboratory provided a landmark observation in the field by showing that neutrophils could make superoxide by the one-electron reduction of oxygen [10, 11]. An important driver of the idea that superoxide may be produced by an oxidase in immune systems stemmed from pioneering work by Irwin Fridovich and colleagues who were studying the actions of xanthine oxidase. They first showed that oxygen-dependent cytochrome C reduction was likely driven by superoxide, conducting electrons from xanthine oxidase to cytochrome C [12]. In support of this, they noted that xanthine oxidase could also cause luminol and lucigenin to luminesce. Moreover, further work suggested the existence of a protein that was capable of catalytically eliminating superoxide, and they went on to identify superoxide dismutase [13]. As they further developed their experimental techniques for preparing large quantities of superoxide, they were able to demonstrate both its ability to reduce cytochrome C and the presence of several superoxide dismutase enzymes in a variety of organisms [14]. This had two important physiological implications: that superoxide could be made in biological systems, and that one function of Fridovich’s newly described bacterially encoded superoxide dismutases might be to degrade superoxide made by the immune system and therefore evade killing [15].

Where do the electrons come from? Evidence was presented for both NADPH and NADH as the donor. Rossi and Zatti’s 1964 publication proved prescient and over the ensuing years, other studies supported growing evidence that NADPH was the electron donor in this context [16]. The description of the biochemistry of the phagocyte NADPH oxidase was a major advance in itself but parallel work on a rare immunodeficiency demonstrated how synergy between discovery science and clinical observation can lead to fundamental advances.

The first descriptions of chronic granulomatous disease (CGD) emerged after discussions at a conference of the American Paediatric Society in 1954 where both Charles Janeway Senior and Robert Good described cohorts of boys with severe and recurrent infections. The initial publication of Good’s and Janeway’s cohorts [17] was followed by the demonstration that leucocytes from affected patients were unable to kill bacteria [18] and did not undergo the changes in respiration that we now know to be associated with phagocyte NADPH oxidase activity [19]. Furthermore, there was clearly defective production of superoxide; patients’ leucocytes failed to reduce nitroblue tetrazolium (NBT) during phagocytosis [20]. This test remains a standard method for assessing oxidase activity. This lack of oxidase activity in patients with CGD gave the clearest indication yet that the purpose of making ROS was for host defence. Subsequent work added to this evidence, showing that cells from CGD patients were unable to make H_2_O_2_ [21] and superoxide [22].

Biochemical and clinical studies converged when work from Tony Segal and colleagues proposed that a novel, membrane-resident b-type cytochrome was responsible for superoxide production and that, crucially, this was not expressed in patients with X-linked CGD (X-CGD) [23]. Any doubt that this was the phagocyte NADPH oxidase was dispelled by two seminal studies. First, the causative genetic region was localised to chromosome Xp21 and cloned [24]. The cDNA identified from these studies was used to make a translated protein and an anti-serum was raised to it. This anti-sera stained a 91kDa protein found in “purified cytochrome b558” preparations and it could *not* stain neutrophils from patients with X-CGD [25]. The unusual cytochrome identified by Segal was indeed the product of the gene that was disrupted in X-CGD. These observations were extended to show that cytochrome b558 is a flavoprotein (FAD-binding) that incorporates two hemes, thus providing all the apparatus necessary for electron transfer to oxygen. It was thus the first flavocytochrome ever described in higher eukaryotic cells.

The electron transfer mechanism utilised by cytochrome b558 to produce ROS can be broken down into distinct steps, reviewed by Cross and Segal [26]:

NADPH accesses its binding site in gp91*phox*, which is made available during the activation mechanism. Two electrons are then transferred to FAD: FAD often takes part in reactions in which two electrons are transferred from a donor but only one is needed, leading to a reduction in target molecules.

FAD transfers an electron to the inner heme molecule. This same electron is transferred to the outer heme, and then on to molecular oxygen. This is then repeated for the second electron bound to FAD, with the difference that FAD is now a semiquinone (a one-electron intermediate), meaning that the driving force for reduction is weaker than in the two-electron condition.

In addition to the elucidation of how structure related to function in gp91*phox*, work in the 1980s showed that it had a heterodimeric membrane binding partner. In 1987, Segal and colleagues refined the technique for isolation of the gp91*phox* subunit. Using more powerful protease inhibitors and a rapid isolation technique, they showed that an additional 22 kDa protein co-purified with gp91*phox* [27], named p22*phox*. The two subunits (gp91*phox* and p22*phox*) were closely linked and remained associated with the heme of the cytochrome through both affinity and gel filtration chromatography and also sucrose gradient centrifugation. At the same time, by refining their own techniques, Parkos and colleagues also demonstrated that gp91*phox* co-purified with a 22kDa protein [28]. Such work laid the foundation for the important principle that gp91*phox* and p22*phox* are a co-dependent heterodimer and genetic deletion of one protein results in proteolytic degradation of its partner. The biochemical description of p22*phox* eventually led to the finding that mutations in the *CYBA* gene, that encodes this protein, can also cause CGD [29]^.^

One persistent question despite the impressive dissection of the nature of gp91*phox* was that there were also autosomal recessive forms of the disease [30–32], and in most of these, the expression of gp91*phox* is normal (hindsight tells us that p22*phox* deficiency will be the exception). Once again, the convergence of biochemistry and careful clinical phenotyping provided answers. Cell free systems proved instrumental in understanding the phagocyte NADPH oxidase better. An excellent review can be found in [33]. Essentially, Edgar Pick’s group was attempting to isolate the membrane fraction of IFN-γ activated macrophages in order to make accurate measurements of superoxide production. In setting up their “cell free system” of homogenates from guinea pig macrophages, they found that they could no longer reconstitute oxidase activity if the homogenate was separated into membrane and cytosolic fractions. Adding the cytosolic fraction back restored the system’s ability to produce superoxide in response to arachidonic acid. This demonstrated that the oxidase was not a single membrane bound entity but had cytosolic components. Other reports on similar cell free systems followed, either contemporaneously or shortly after [34–36]. One report also made the prescient observation that components from the cytosolic fraction translocated to the membrane [35].

Volpp et al [37] modified the cell-free technique based on the observation that the oxidase activity of cell-free systems was augmented by the addition of GTP. They purified such proteins by GTP-agarose affinity chromatography and showed that the superoxide-generating capacity of the cytosol resided in the GTP-bound fractions. Raising anti-sera against the proteins in the “active” part of the cytosol, they found that they bound two proteins of approximately 47kDa and 67kDa. Crucially, either p47*phox* or p67*phox* was absent in some cases of autosomal recessive CGD, confirming the role of these proteins as integral cytosolic components of the NADPH oxidase complex. Another paper also utilizing cell-free systems, published in the same issue of *Science*, demonstrated very similar results [38]. This elaborated on a previous observation by Segal that neutrophils from patients with a form of CGD failed to phosphorylate a 44kDa protein (p47*phox*, it transpired). The success of these approaches rests on the observation that GTP augments the activity of the oxidase and, therefore, GTP-binding proteins must be involved. Indeed, it was shown that a GTP binding protein (originally defined as sigma-1) was required for superoxide production in the cell-free system [39]and this was subsequently identified as a heterodimer of Rac1 and the GDP-dissociation inhibition factor (GDI) [40, 41].

Almost contemporaneous with the description of Rac1, Knaus and colleagues used a very similar experimental design to isolate a GTP-binding protein that could facilitate activation of the oxidase in cell-free systems [42]. They noted that GDP and inhibition of protein isoprenylation inhibited the process. This latter point was important because a post-translational modification that attaches 15-20 carbon isoprenoid moieties is a common feature of GTP-binding proteins of the Ras superfamily. They identified Rac2, rather than Rac 1, as the relevant GTP-binding protein. This reflects the fact that Abo et al [41] used macrophages for their assays whereas Knaus et al used neutrophils. Much subsequent work has shown that Rac1 is the predominant form used in macrophages, whereas neutrophils use Rac2 by preference. Rac1 deficiency is embryonically lethal in mice [43]. Human mutations that lead to impaired Rac2 activity have been described and are more complex than classical CGD, reflecting the role of the protein in a wide variety of neutrophil functions [44, 45], including chemotaxis [46]. Meanwhile, the cell-free system would advance such that the cytosolic factors could be replaced by three recombinant proteins, p47*phox*, p67*phox,* and Rac1, thus describing the minimal essential requirement of cytosolic factors for production of superoxide in the cell-free system [47].

Prompted by the fact that p67*phox* and p47*phox* were part of a complex of approximately 250kDa on gel filtration chromatography, Wientjes et al identified p40*phox* as a new cytosolic component of the complex, but **not** one that is necessary for superoxide production in the cell-free system [48]. Nevertheless, p40*phox* is essential for activation of the oxidase in certain cellular contexts. This is illustrated by mouse studies that showed p40*phox*^-/-^ mice are susceptible to Staphylococcus aureus infection and that p40*phox* deficiency can cause CGD [49, 50], albeit with a rather different phenotype to classical CGD. This is discussed in more detail below.

## NADPH oxidase: structure and activation

### Structure of the cytosolic components and their interactions

A brief description of the cytosolic subunits p67*phox*, p47*phox*, p40*phox*, is provided in Table 1, including mention of the Phox homology (PX) domain and the Phox and Bem1 (PB1) domain, both domains first identified in these proteins [65, 66].

**Table 1:** cytosolic components of the NADPH oxidase

	MW (aas)	Important domains	Interactions (activated)	Interactions (inhibited)
p47*phox*	47 kDa (390)	PX SH3 ^*^2 Polybasic region Proline rich region	PX—binds to PtdIns(3,4)P2 located in membranes [51] Two SH3 domains—bind to cytoplasmic tail of p22*phox* [52]	Autoinhibition - SH3 domains bind to polybasic region [53] Phosphorylation of serines relieves autoinhibition [54, 55]
p67*phox*	67 kDa (526)	TPR ^*^4 PRR SH3 PB1 SH3 ‘Activation domain’—aas 199-210	PB1—creates a heterodimer with PB1 in p40*phox* [56] Four TPR domains in N terminal—bind to Rac proteins 1:1 [57] ‘Activation domain’—possible direct interaction with flavocytochrome, regulate electron transfer [58, 59]	Possible autoinhibition relieved by phosphorylation of Threonine 233 [58]
p40*phox*	39 kDa (339)	PX SH3 PB1	PX—binds to PtdIns(3) located in phagosomal membranes [60] SH3—may bind to PRR in p47*phox* [61] PB1—binds to p6*7phox* [56]	Expression may be stabilized by p67*phox* [62–64]

^*^PX -Phox homology domain, SH3—Src homology 3, TPR—tetratricopeptide repeat, PRR—proline rich region, PB1—Phox and Bem1 domain, PtdIns—Phosphatidylinositol.

In the resting state, p40*phox*, p47*phox* and p67*phox* associate with a 1:1:1 stoichiometry [67, 68]. The high molecular mass of 250kDa previously ascribed to these three proteins in complex is likely attributable to the non-globular shape of the complex [68].

### Assembly and activation of the phagocyte NADPH oxidase

Several events must take place to facilitate assembly of the multi-subunit complex and electron transfer. A few key principles of the process are set out below. In the resting state, the gp91*phox*-p22*phox* heterodimer is sequestered away from the p47*phox*-p67*phox*-p40*phox* cytosolic subunits [69]. Numerous stimuli can activate the oxidase, and among the best known include protein kinase C stimulators such as phorbol myristate acetate (PMA), the bacterial peptide fMLP, opsonised zymosan, opsonized bacteria, and the complement fragment, C5a. The formation of the active complex is prevented in the resting state by:

(i) Physical separation of the membrane-bound and cytosolic components
(ii) The requirement for post-translational modifications such as phosphorylation of the subunits
(iii) Rac proteins in an inactive state.

A key early event is phosphorylation of serines on p47*phox* that releases it from auto-inhibition and facilitate activation of the NADPH oxidase. Combined mutations show of the C-terminal serines shows that they are required for activation of the oxidase driven by fMLP, PMA, and IgG [55], while individual mutation of serines shows that only phosphorylation of serine 379 is absolutely required for efficient activation [54]. The effect of serine phosphorylation on p47phox is to enhance the p22*phox*-p47*phox* interaction and also the binding of p67*phox* to gp91*phox* [58, 70] (see below). Neither p40*phox* nor p67*phox* is able to translocate in the absence of p47*phox*, as evidenced by the cytoplasmic location of p40*phox*–p67*phox* in stimulated cells from CGD patients that lack a functional p47*phox* [62]. Docking of the p47*phox*–p67*phox*–p40*phox* complex to the membrane bound cytochrome *b*558 is supported by an interaction between the tandem SH3 domains of p47*phox* and the cytoplasmic tail of p22*phox*. The crystal structure revealed that both SH3 domains co-operate to mediate this interaction [68].

During activation, p22*phox* also becomes phosphorylated [71]. Several stimuli that drive the respiratory burst in neutrophils, such as fMLP, PMA, and opsonized zymosan, lead to its phosphorylation by both phospholipase D dependent and independent mechanisms [72]. The major site of phosphorylation is threonine 147 and mutation of this residue to an alanine inhibits the interaction between p22*phox* and p47*phox* and activation of the oxidase [73].

Turning our attention to p67*phox* and Rac, p67*phox* must interact with GTP-bound Rac for activation to proceed. This occurs after both proteins have, independently from one another, translocated to the membrane. The likely role of Rac is both to tether p67*phox* to the membrane and induce a conformational change. These two proteins, together with the cytochrome b558, are sufficient to induce electron transfer in cell free systems, although significantly higher concentrations of either are required in the absence of p47*phox* [74, 75]. p47*phox* is not, therefore, a *sine qua non* for the process [74] but acts as an “organiser” subunit that binds to p22*phox* and forms a bridge between p22*phox* and p67*phox*. It also binds to the cytoplasmic domain of gp91*phox*, stabilising the interaction between cytochrome b558 and p67*phox*. p67*phox* contacts gp91*phox* via its activation domain, and this is essential for mediating electron transfer across gp91*phox* in a cell-free system [76, 77]. The association of recombinant p67*phox* with flavocytochrome b558 in liposomes also induces conformational changes that can be detected by atomic force microscopy [78]. Thus, translocated p67*phox* may mediate allosteric effects on the catalytic activity of flavocytochrome b558.

## Recent advances in understanding of the phagocyte NADPH oxidase

### Discovery of EROS

Recently, we described a new component critical to NADPH oxidase function, designated EROS (Essential for Reactive Oxygen Species, gene symbol, *CYBC1*) [79,80]. Work on the function of this gene stemmed from the observation that mice deficient in the previously undescribed open reading frame, *bc017643*, were highly susceptible to infection with *Salmonella* Typhimurium [79,80]. In C57BL/6 mice, effective production of ROS is necessary to control Salmonella Typhimurium, and the *bc017643*^−/−^ mice made very little ROS, either in neutrophils or macrophages. It transpired that while the mRNA abundance of *CYBB* and *CYBA* (the genes encoding gp91*phox* and p22*phox*) were normal, very little gp91*phox* and p22*phox* was expressed in mice lacking the gene. We thus re-named *bc017643* as EROS (Essential for Reactive Oxygen Species). EROS co-immunoprecipitated with gp91*phox* and co-localised with it in the endoplasmic reticulum. We hypothesised that the protein was likely a chaperone for the complex, likely binding gp91*phox* [79,80]. The mouse gene had a highly similar human ortholog *C17ORF62,* and we and others found mutations in this gene could cause CGD in humans (see below) [79,80]. The gene name was therefore changed to *CYBC1* (cytochrome B chaperone 1).

We have now shown that EROS is indeed a chaperone [81], but a highly selective one that controls the abundance of only a few proteins. It binds directly to the translated precursor of gp91*phox* and remains associated with it throughout glycosylation and heme insertion before “handing it over” in its mature form to p22*phox*. The evidence for direct interaction came from both yeast 2-hybrid and split luciferase “nanobiT” assays, and we also showed that EROS, gp91*phox,* and p22*phox* could be found in a complex by size exclusion chromatography. In this paper, we also made use of co-transfection assays in which gp91*phox* abundance and stability could be hugely augmented by co-expression with EROS. It was noticeable however, that EROS co-transfection specifically augmented expression of the immature 58kDa gp91*phox* precursor. EROS co-transfection had no effect on p22*phox* abundance. These experiments complimented those from the laboratories of Mary Dinauer and William Nauseef some 20 years previously which showed that co-transfection of p22*phox* with gp91*phox* led to augmented gp91*phox* expression, specifically the mature form

Interestingly, in our initial mass spectrometry analysis of EROS sufficient and deficient macrophages, we noted that P2X7 was also highly down-regulated on EROS^−/−^ macrophages [ [79]]. P2X7 is an ATP-gated cation channel that binds extracellular ATP, permitting Na + and Ca2 + influx and K + efflux. It is a major driver of NLRP3 inflammasome activation as well as several other processes, including ROS production and NF-κB activation [82]. This phenomenon was independently shown by another group, who showed that EROS controlled P2X7-mediated phosphatidylserine excursion [83]. This control of P2X7 illustrates that proteins such as EROS can moonlight and have several distinct functions.

### Structural resolution of NADPH oxidase components

Recently, two landmark studies have significantly advanced our understanding of cytochrome b558 assembly by resolving the cryo-EM structure of gp91*phox* and p22*phox* in complex [84, 85]. These studies show that, as expected, gp91*phox* has 6 transmembrane domains. It also showed that p22*phox* has 4 transmembrane domains and that an extensive interface is formed between gp91*phox* and p22*phox* based on electrostatic interactions and shape complementarity. Both papers define the interface as TM3–TM4–TM5 of NOX2 and TM1 and TM4 of p22*phox*. Liu et al describe a 9618A interface between gp91*phox* and p22*phox*, their complementary shapes, and describe key interacting residues between the two, including residues in gp91*phox* which are conserved in NOX1-4. Noreng et al also describe the transmembrane interface as well as interactions on the extracellular side between NOX2 TM3-loop C/p22 ECL1-ECL2 and the cytoplasmic side: NOX2 TM4-loop D-TM5/p22 ICL1-TM1.

Certain parts of both proteins are less well visualized as they are likely stabilised by interactions with cytosolic factors. For instance, in the publication by Noreng et al in gp91*phox* [84], the heme-binding ferric oxidoreductase transmembrane domains and the three extracellular loops are well-resolved, whereas the intracellular FAD and NADPH-binding dehydrogenase domain (DHD) is only visualised at lower cryo-EM map contours. Similarly, in p22*phox*, resolution of the intracellular C-terminus, containing the proline-rich region involved in p47*phox* binding, is disordered and less well resolved than the transmembrane domains and intracellular loops.

### Structural resolution of EROS binding to gp91*phox*

A key question following from this is where EROS binds gp91*phox*, based on observations from our group that the EROS gp91*phox* interaction is direct. This question was recently addressed in an elegant study from Liang and colleagues who resolved the cryo-EM structure of the EROS-gp91*phox*-p22*phox* complex to 3.56A [86]. Within the membrane, the two transmembrane helices of EROS interact with transmembrane helices 2 and 6 of gp91*phox*. An intracellular view of the complex shows that EROS and p22*phox* are on opposite sides of it with a plane angle of 177 degrees. This fits with our previous observations that EROS is unable to augment p22*phox* abundance in co-transfection assays in the same way that it does for gp91*phox* and that EROS and p22*phox* do not interact in yeast two-hybrid assays. Similarly, in our initial publication on EROS [80] we found that p22*phox* was quite rescuable with proteasome inhibition whereas gp91*phox* was not, implying that the primary defect in EROS deficiency lay in abnormal gp91*phox* folding and effects on p22*phox* were secondary.

The paper by Liang et al. describes three interactions between EROS and gp91*phox* that may impede electron transfer.

1. EROS contacts the inner heme of gp91*phox* and the presence of EROS shifts the heme’s positions downwards.. This serves to lengthen the distance between the inner and outer hemes,
2. There are multiple hydrophobic interactions between the β-strands of EROS and FAD-binding domain of NOX2,forming a tight binding interface, preventing FAD binding It is worth noting that mutations of residues 338, 339 and 341 in gp91*phox*, located in the FAD-binding domain, cause CGD.
3. There is a region of contact with EROS in in the subdomain of gp91*phox* that binds NADPH. The affinity of resting NOX2 for NADPH is lower than that for FAD. Thus EROS binding may impair NADPH binding.

The regulation of electron transfer by EROS in the endoplasmic reticulum makes sense as one would not want electron transfer to occur in the wrong compartment during assembly of the gp91*phox*-p22*phox* heterodimer. A key question then, is whether EROS can ever accompany the mature gp91*phox*-p22*phox* heterodimer through the Golgi apparatus and to compartments such as the plasma membrane, neutrophil granules and endosomes where phagocyte NADPH oxidase effector function takes place. If that was the case, one could envisage situations in which it is a true physiological regulator of oxidase activity by remaining bound to the complex and inhibitory to electron transfer. The paper by Liang and colleagues suggests that a small proportion of EROS can localise to the cell membrane, though this analysis used a polyclonal antibody. It would be ideal to verify the specificity of cell surface localisation of EROS seen in flow cytometry and confocal microscopy by evaluating the same primary antibody and secondary antibody pair on cells that are EROS deficient.

Nevertheless, if EROS can get to the plasma membrane to regulate oxidase activation, this is an important point and raises the question of what governs association and dissociation of EROS. Are these the same physiological stimuli (e.g. zymosan and fMLP) and enzymes (e.g. protein kinase C) that are known to drive activation of the phagocyte NADPH oxidase? The study from Richard Ye’s group [86] made some preliminary advances here by using the nanobIT system that we had also deployed in our publication [81] to show that protein kinase C stimulation by PMA may promote this dissociation.

This work also suggests that an alternative form of EROS-driven inborn error of immunity could potentially exist in which “dominant negative” mutations of EROS prevent dissociation from gp91*phox* even with stimulation and lead to impaired NADPH oxidase activation. In such cases, gp91*phox* and p22*phox* abundance would likely be normal but the enzyme would be non-functional.

Moreover, we have also shown that EROS is expressed in non -haematopoietic tissues such as endothelial cells [87], where it has functions distinct from those we have observed in the immune system. In the tissues we have studied (kidney, endothelium, colon), EROS does not regulate the abundance of NOX1 or NOX4 (which also depend on p22*phox* for stability) but it could regulate their function if it binds them in the same way as that proposed for gp91*phox* and is able to influence the process of electron transfer.

### Structural resolution of activated phagocyte NADPH oxidase

Our understanding of the structural basis of NADPH oxidase activation has advanced even further recently with the first cryo-EM structure of the membrane bound components together with fragments of the cytosolic components p47*phox*, p67*phox* and Rac1 [88]. This paper makes a case for having captured the phagocyte NADPH oxidase in the activated state. A number of factors suggest that this structure represents some physiological version of the activated phagocyte NAPDH oxidase including the fact that the cryo-EM preparation, consisting of a ternary complex of NOX2, p22*phox*-GFP-p67*phox* and prenylated GFP-p47*phox*-Rac1, has oxidase activity. Similarly, an NADPH substrate density was observed in some nanodisc preparations of the “activated” state and this is not seen in the “resting” structure of the phagocyte NADPH oxidase in contrast to FAD which seems to be present in both states.

The key structural insights from this paper revolve around how the cytosolic factors facilitate more efficient electron transfer when they contact the membrane heterodimer. The binding of the p67*phox*–Rac1 complex induces the contraction of the dehydrogenase (DH) domain and the docking of the DH domain to the transmembrane domain (TMD). This stabilizes the binding of NADPH and allows the efficient transfer of electrons from intracellular NADPH to extracellular oxygen. The contraction of the DH domain also decreased the distance between the flavin binding domain (FBD) and the nucleotide binding domain (NBD). This would theoretically increase the rate of electron transfer from NADPH to FAD. This is consistent with previous work that reported that the binding of p67*phox*–Rac1 to gp91*phox* enhances the rates of electron transfer from NADPH to FAD. Finally, in the activated state, the docking of the dehydrogenase domain onto the bottom of the transmembrane domain of NOX2 reduces the distance between FAD and the inner haem. These changes in the “activated” state to drive electron transfer provide an interesting contrast with those described for the EROS-bound structure, which seem to actively prohibit it.

## Chronic granulomatous disease: mutations affecting NADPH oxidase activity

### Current understanding of “classical” chronic granulomatous disease

Chronic granulomatous disease results from mutations affecting different components of the NADPH oxidase and its ROS production. The different subtypes are summarised below [table 2], and an updated list of verified pathogenic mutations can be found in this publication [89].

**Table 2:** the subtypes of CGD

Gene (inheritance)	Protein affected	Relative frequency [90]	Geographical distribution	Infectious phenotype	Inflammatory phenotype
*CYBB* *(x-linked)*	gp91*phox*	~66%	Most common mutation in China [91, 92], Europe [93], USA [94]	Invasive bacterial and fungal infections [95, 96] Female carriers of x-linked mutation: invasive infections when skewed X-inactivation [97]	·Cutaneous manifestations [98] ·Granulomatous colitis ·Urinary tract inflammation ·Lung inflammation [89] · Female × linked carriers: ANA—negative lupus- like syndrome [99]
*CYBA (AR)*	p22*phox*	7%	AR mutations are most common in Jordan, Syria and Iraq [100], India [101, 102] Turkey [103]		
*NCF2 (AR)*	p67*phox*	7%			
*NCF1 (AR or unequal recombination)* [104]	p47*phox*	20%			
*NCF4 (AR)*	p40*phox*	Rare		Non-invasive infections [50]	
*CYBC1 (AR)*	EROS	Rare		Invasive infections [79]	Granulomatous inflammation (as above) but also: Glomerulonephritis [105] Haemolytic anaemia [79]

The epidemiology and incidence of CGD is well studied and the disease is found in about 1/200,000 live births [89]. Current studies describe the majority of CGD cases as caused by X-linked mutations while mutations in the autosomal components of the NADPH oxidase complex account for approximately 33% of disease cases [90]. The autosomal recessive forms of the disease are found more commonly in parts of the world where consanguineous marriage is common and, as more cohorts of patients with CGD are analysed worldwide, the ratio of X-CGD to AR-CGD might change. Residual ROS production correlates strongly with survival in CGD [106]. Mutations leading to impaired intracellular superoxide production in neutrophils with preserved extracellular release of ROS, as in the less common *NCF4* (p40*phox*)-deficient CGD, lead to milder infectious manifestations with more pronounced hyperinflammation syndromes [50].

Hyperinflammatory manifestations in CGD, evidence of the many roles of ROS in the immune system (Table 3), do not seem to be related to residual oxidase activity, in contrast to the risk of developing infectious manifestations [94, 99]. This is demonstrated in X-linked CGD, in which the heterozygous female relatives of affected males have a proportion of innate immune cells that are fully oxidase-deficient. In this group even a small subset of cells that do not possess oxidase activity appear to be able to drive autoinflammation and autoimmune manifestations in a dominant fashion. The most common presentation is a lupus-like syndrome that is often anti-nuclear antibody negative but which can present with positive lupus serology. Several cohorts have been described at a variety of centres expert in treating CGD [94, 98, 99, 118].

**Table 3:** immune processes affected by ROS

Type 1 interferon (IFN-I)	•Increase in STAT1 transcription in ROS deficiency [107] •Elevated IFN-I signalling genes in ROS deficiency [108]
**Inflammasome**	•Uncontrolled inflammasome activation in ANCA vasculitis with ROS deficiency [109] •Increased levels of caspase-1 and IL-1b in CGD [110]
**NF-kB signalling**	•Lack of oxidation of a cysteine in NF-κB subunit p50 leading to overactivation in ROS deficiency [111] •Lack of glutathionylation in the inhibitor IκB leading to its phosphorylation and degradation in ROS deficiency [112]
**Autophagy**	•ROS important for pathogen engulfment via LC3-associated phagocytosis [113, 114], a form of non-canonical autophagy.
**Antigen presentation**	•ROS regulate MHC class I presentation via oxidative stress in endosomes leading to pathogen leak [115] •May regulate MHC class I via cysteine-containing cathepsins [116] •Regulate MHC class II presentation [117]

### A new cause of CGD

Recently, we described a 5th cause of CGD: EROS (*CYBC1*) deficiency [79].In 2018 both we and an Icelandic group [105] showed that mutations in *CYBC1 could* cause CGD. Interestingly, there are eight cases in Iceland and this relates to the fact that 1/70 people there are heterozygous for a mutation in *CYBC1* that introduces a stop codon after the second amino acid.

It remains to be seen how much EROS (*CYBC1*) deficiency recapitulates “classic CGD’ as there are, as yet, only a handful of cases described. Sterile granulomatous inflammation and opportunistic infection have both been described, but so have other manifestations not associated with CGD, such as autoimmune haemolytic anaemia [79]. As previously mentioned, we have now shown that EROS is a highly selective chaperone of gp91*phox* [81], but also controls P2X7, a major driver of NLRP3 inflammasome activation and NF-κB signalling. Thus, it is likely that *CYBC1* deficiency will have some distinct features from other forms of CGD.

EROS may play a role in other, classical forms of CGD. New mutations in *CYBB* are still being described [119] and it will be interesting to investigate if any of the missense mutations in gp91*phox* disrupt the binding site with EROS, preventing early stabilisation of the gp91*phox* through its maturation.

### Other deficiencies affecting NADPH oxidase function leading to autoimmunity

It has also become clear that some clinical manifestations of other immunodeficiencies may relate to the involvement of the causative gene in the respiratory burst. For instance, Protein kinase C delta deficiency was first reported as a cause of primary immunodeficiency in 2013 [120, 121]. Recently, an international consortium re-visited disease mechanisms in this cohort [122]. They noted that the clinical syndrome had many features reminiscent of CGD, including monogenic systemic lupus erythematosus and infections with *Staphylococcus* and *Pseudomonas* species. They found a severely impaired respiratory burst in response to PMA and Staphylococcus aureus from both immortalised B cells and phagocytes from the patients, and this was associated with defective phosphorylation of p40*phox.*

It is noteworthy that in syndromes secondary to dysfunction of EROS or protein kinase C, the loss of the oxidative burst is not complete and may be partial or cell type specific to some extent. This suggests that there may be other undiscovered regulators of the process and that some known inborn errors of immunity are due in part to defective ROS generation but this may be missed by “all or nothing” assessment of ROS generation by nitroblue tetrazolium (NBT) or dihydrorhodamine (DHR) tests in response to a limited range of stimuli. It is likely there is still much to learn about the layers of regulation needed to maintain efficient control of the phagocyte NADPH oxidase.

## Concluding remarks

In this review, we have outlined the key discoveries in chronic granulomatous disease and phagocyte NADPH oxidase biology. The disease is an excellent example of how careful dissection of cell biology has led to a deeper understanding of disease and new insights continue to be made at regular intervals. The ways in which polymorphisms in the NAPDH oxidase subunits affect the risk of common autoimmune diseases is also a fascinating area. There is much still to do for patients with CGD: importantly, gene therapy and gene editing technologies coupled with improvements in pre-transplant management [123, 124] mean that the future looks much brighter for patients than it did thirty years ago.

## Data Availability

Not applicable.

## Abbreviations

CGD: chronic granulomatous disease;
H_2_O_2_: hydrochloric acid;
NOX2: phagocyte NADPH oxidase;
ROS: reactive oxygen species;
X-CGD: X-linked chronic granulomatous disease
